# Stat6-Dependent Inhibition of Mincle Expression in Mouse and Human Antigen-Presenting Cells by the Th2 Cytokine IL-4

**DOI:** 10.3389/fimmu.2016.00423

**Published:** 2016-10-14

**Authors:** Thomas Hupfer, Judith Schick, Katrin Jozefowski, David Voehringer, Jenny Ostrop, Roland Lang

**Affiliations:** ^1^Institute of Clinical Microbiology, Immunology and Hygiene, Universitätsklinikum Erlangen, Friedrich-Alexander-Universität Erlangen-Nürnberg, Erlangen, Germany; ^2^Department of Infection Biology, Universitätsklinikum Erlangen, Friedrich-Alexander Universität Erlangen-Nürnberg, Erlangen, Germany; ^3^Department of Cancer Research and Molecular Medicine, Centre of Molecular Inflammation Research, Norwegian University of Science and Technology (NTNU), Trondheim, Norway

**Keywords:** C-type lectin receptor, Mincle, Mcl, IL-4, macrophage, monocyte

## Abstract

The C-type lectin receptors (CLRs) Mincle, Mcl, and Dectin-2 bind mycobacterial and fungal cell wall glycolipids and carbohydrates. Recently, we described that expression of these CLR is downregulated during differentiation of human monocytes to dendritic cells (DC) in the presence of GM-CSF and IL-4. Here, we demonstrate that the Th2 cytokine IL-4 specifically inhibits expression of Mincle, Mcl, and Dectin-2 in human antigen-presenting cells (APC). This inhibitory effect of IL-4 was observed across species, as murine macrophages and DC treated with IL-4 also downregulated these receptors. IL-4 blocked upregulation of Mincle and Mcl mRNA expression and cell surface protein by murine macrophages in response to the Mincle ligand Trehalose-6,6-dibehenate (TDB), whereas the TLR4 ligand LPS overcame inhibition by IL-4. Functionally, downregulation of Mincle expression by IL-4 was accompanied by reduced cytokine production upon stimulation with TDB. These inhibitory effects of IL-4 were dependent on the transcription factor Stat6. Together, our results show that the key Th2 cytokine IL-4 exerts a negative effect on the expression of Mincle and other Dectin-2 cluster CLR in mouse and human macrophages and DC, which may render these sentinel cells less vigilant for sensing mycobacterial and fungal ligands.

## Introduction

Following the identification of Dectin-1 as a Syk-coupled activating receptor for bacterial and fungal beta-glucans 15 years ago (1), C-type lectin receptors (CLRs) have received increasing attention as pattern recognition receptors of the innate immune system. The CLR Dectin-2, Mincle, and Mcl belong to the so-called Dectin-2 family with their genes adjacent to each other in the NK cell receptor gene cluster, localized on human chromosome 12 and mouse chromosome 6 (2). These three receptors recognize mycobacterial and/or fungal ligands, are expressed constitutively or inducibly on innate immune cells, and have an emerging role in innate immunity to these infections.

Mincle (official gene symbol *Clec4e*, aka *Clecsf9*) not only binds the mycobacterial cord factor trehalose-6,6-dimycolate (TDM) (3, 4), related synthetic glycolipids such as the adjuvant trehalose-6,6-dibehenate (TDB) (3, 4), but also fungal ligands derived from *Candida albicans* (5–7), *Malassezia furfur* (8, 9), or *Fonsecaea* spp. (10, 11). More recently, Mcl (official gene symbol *Clec4d*, aka *Clecsf8*) was identified as a second, low affinity-receptor for the mycobacterial cord factor (12), which is required for efficient control of experimental mycobacterial infection in mice (13), but did not bind to sugar ligands on carbohydrate microarrays (14). In contrast, Dectin-2 (gene symbol *Clec4n* in mice, *CLEC6A* in humans) not only has a classical C-type lectin domain that binds structures with high mannose content from numerous pathogens, most notably *Candida albicans* (15, 16), but also mycobacterial manLAM (17) and schistosomal egg antigen (18).

Whereas Dectin-1 directly recruits the kinase Syk *via* a non-classical ITAM motif in its intracellular domain, Mincle, Mcl, and Dectin-2 all associate with the ITAM-containing adapter protein Fc receptor gamma chain (FcRγ) to initiate signaling through the Card9/Bcl10/Malt1 complex (19). Activation of NFκB and MAPK pathways triggers substantial reprograming of gene expression in macrophages after activation of Mincle by TDB, similar to Curdlan-induced Dectin-1 activation, but only partially overlapping with inflammatory gene expression induced by TLR ligands (20). Similar to Curdlan, activation of APC by TDB or TDM directs a cytokine milieu fostering the development of Th17 responses to co-delivered protein antigens *via* production of IL-6, IL-23, and IL-1 (4, 20–24).

Expression of Mincle is strongly inducible in murine macrophages and DC by PAMPs, such as the TLR4 ligand LPS (25) or by its ligand TDM itself (4, 12), and depends on the transcription factor C/EBPβ (25, 26). Interestingly, Mincle expression is constitutively high in murine monocytes and granulocytes (21, 27), similar to human monocytes and macrophages (28). In contrast, expression of Mcl is constitutively higher in mouse bone marrow-derived macrophages (BMM) and bone marrow-derived dendritic cells (BMDC), and inducible to a lesser extent (12, 26). Dectin-2 expression is predominantly myeloid restricted and upregulated by inflammatory stimuli (16). Cytokines involved in the upregulation of Dectin-2 include TNF or GM-CSF (29).

IL-4 is the prototypical Th2 cytokine and induces alternative macrophage activation through the transcription factor Stat6 (30). Interestingly, Th2 responses and IL-4 driven alternative macrophage activation have been associated with poorer outcomes in fungal (31) and in mycobacterial infection (32, 33). It is well established that IL-4 induces the expression of Dectin-1 (34). Its effects on the expression of other CLRs are not well characterized, although downregulation of Dectin-2 expression in IL-4 treated human CD14^+^ monocytes has been described (29).

We recently observed a strong downregulation of the mRNA expression of Mincle, Mcl, and Dectin-2 during differentiation of human DC from CD14^+^ monocytes in the presence of GM-CSF and IL-4 *in vitro* (28). Here, we investigated the regulation of expression of these CLRs by IL-4 in human and mouse APC. Our data show that IL-4 specifically downregulates Mincle, Mcl, and Dectin-2 expression, but not Dectin-1 expression, in both species and impairs Mincle-dependent macrophage activation in response to the cord factor analog TDB.

## Materials and Methods

### Isolation and Culture of Human Antigen-Presenting Cells

The use of human leukocytes from healthy donors with written informed consent complies with the Declaration of Helsinki (Ethical committee Erlangen approval no. 4055 and no. 111_12 B). PMBCs were obtained from leukoreduction system chambers by density centrifugation (35). Monocytes were positively selected from PBMC using α-CD14 microbeads (Miltenyi Biotec), purity was ≥90%. For culture, RPMI1640 was supplemented with 10% (v/v) fetal calf serum (FCS, Biochrom) and 100 U/ml penicillin and 100 μg/ml streptomycin (cRPMI). A total of 50 U/ml GM-CSF (Genzyme) or 50 U/ml M-CSF (Peprotech) were added for differentiation of macrophages. 50 U/ml GM-CSF and 250 U/ml IL-4 (Peprotech) were added for differentiation of DC. Cells were cultured at a density of 0.8 × 10^6^ cells/ml (GM-CSF macrophages, DC) or 1.6 × 10^6^ cells/ml (M-CSF macrophages) for 6–7 days without change of media at 37°C with 5% CO_2_ humidified air.

### Isolation and Culture of Mouse Macrophages and DC

C57BL/6, *Clec4e*^−/−^, Balb/c, and *Stat6*^−/−^ mice were bred at the Präklinische Experimentelle Tierzentrum of the Medical Faculty of the Friedrich-Alexander-University Erlangen-Nürnberg. Mice were housed and humanely sacrificed according to regional government regulations. Bone marrow cells from femurs and tibiae were differentiated to macrophages by culture in complete Dulbecco’s Modified Eagle Medium containing 10% FCS, antibiotics, and 50 μM β-mercaptoethanol (cDMEM) plus 10% L929-cell conditioned medium as a source of M-CSF, as previously described (36). On day 7, adherent macrophages were harvested by Accutase (Sigma) treatment, washed and counted. For generation of BMDC, bone marrow cells were cultured in cDMEM containing 10% conditioned medium of X63 cells producing GM-CSF for 7–8 days before harvesting. Peritoneal exudate cells (PEC) were obtained on day 4 after injection of mice with 2 ml of 4% thioglycollate by flushing the peritoneal cavity with 10 ml of ice-cold PBS.

### Cell Stimulation

Human antigen-presenting cells were stimulated by adding M-CSF (Peprotech, 50 U/ml), GM-CSF (Genzyme, 50 U/ml), or IL-4 (Peprotech, 250 U/ml unless otherwise stated) to the cell culture medium before plating of cells into 48 well cell-culture plates in a concentration of 0.85 × 10^6^/ml. Mouse antigen-presenting cells were stimulated with 10 ng/ml IL-4 (Peprotech), 10 ng/ml LPS (*E. coli* serotype O55:B5, Sigma) by adding the substance to the cell culture medium before plating of the cells into 24 well cell-culture plates in a concentration of 0.5 × 10^6^/ml. TDB (Polar Avanti, 5 μg/ml) was used plate-bound as previously described (4, 37), coating with isopropanol only was used as a negative control.

### mRNA Expression Analysis of CLRs

RNA was isolated with Trifast (Peqlab), cDNA was transcribed using a cDNA synthesis kit (Applied Biosystems). Expression levels of the housekeeping genes hypoxanthine-guanin-phosphoribosyltransferase (*Hprt*, mouse) or cyclophilin A (*PPIA*, human), as of the genes of interest, were analyzed using primer/probe combinations selected from the Roche Universal Probe Library (Roche). For human MINCLE, the primer/probe combination was selected using the software PrimerExpress (Applied Biosystems). All primers and probes used have been described (28) and were purchased from Metabion. ΔCT values were calculated as ΔCT = CT_(housekeeping gene)_ − CT_(gene of interest)_ (such that higher values indicate higher relative expression), and ΔΔCT values referred to the calibrator as indicated.

### Cytokine ELISA

Cytokine concentration of murine G-CSF and TNF-α was analyzed by sandwich ELISA (DuoSet ELISA, R&D Systems) using cell-free cell culture supernatants of cells stimulated as indicated.

### Flow Cytometry of Mincle and Mcl Surface Receptor Levels

The 2 × 10^5^ BMM of C57BL/6 and Mincle−/− mice were stimulated with LPS (10 ng/ml) or TDB (5 μg/ml) in the presence or absence of IL-4 (10 ng/ml), as well as with IL-4 alone, for 16 h in F-bottom 96 well cell-culture plates. Cell surface expression of Mincle and Mcl was analyzed by flow cytometry. Staining was performed by using anti-Mincle (clone 4A9, MBL) and anti-Mcl (clone 3A4) (38) as primary antibodies, and anti-rat IgG1 conjugated to APC as secondary antibody (eBioscience) in a final concentration of 1 μg/ml for anti-Mincle and anti-rat IgG-APC or 2.27 μg/ml for anti-Mcl. Fc receptors were blocked by adding anti-mouse CD16/32 (clone 93, eBioscience) in a final concentration of 2.5 μg/ml before staining. Cells were stained with primary antibodies for 20 min at 4°C, washed, and then stained with secondary antibody for 20 min at 4°C. Flow cytometry data were acquired on a *FACSCanto II* instrument (BD); analysis was carried out using FlowJo (version 10).

### Statistics

Statistical analysis was performed using Prism5 (GraphPad Software, v5.01). Student’s *t*-test was applied as indicated for non-paired and paired testing between two groups. *p < 0.05, **p < 0.01, ***p < 0.001, ns *p* > 0.05.

## Results

### IL-4 Downregulates MINCLE Expression in Human Monocyte-Derived APC

**Figure 1:**
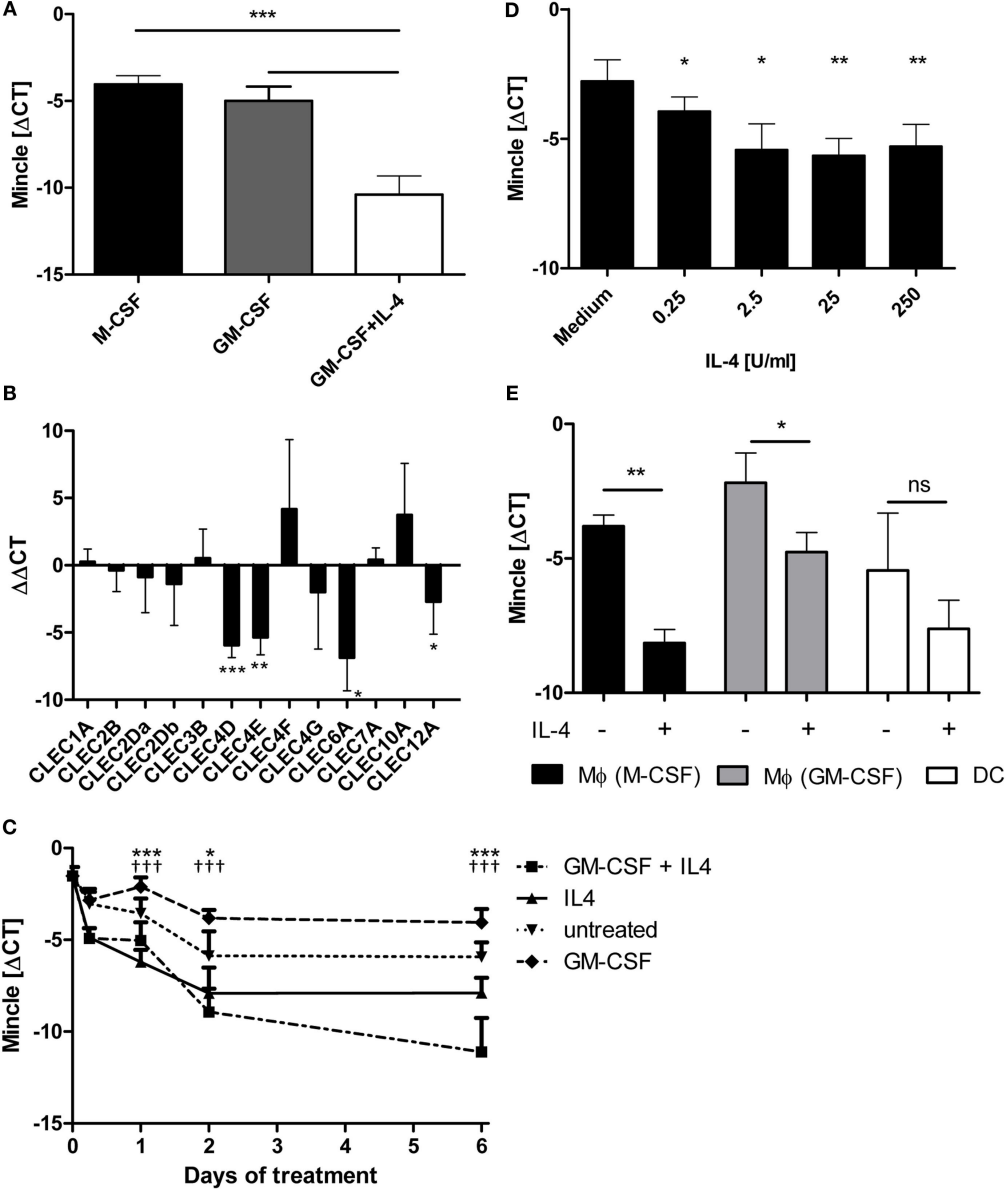
**IL-4 downregulates Mincle mRNA expression in human antigen-presenting cells**. **(A)** Monocyte-derived DC express less MINCLE than monocyte-derived macrophages. Human CD14^+^ cells were differentiated for 6 days in the presence of M-CSF or GM-CSF (to obtain macrophages) or GM-CSF + IL-4 (to obtain dendritic cells). After that, MINCLE mRNA levels were measured using qPCR. The data are depicted as mean ± SD of ΔCT values (Ct_[PPIA]_ − Ct_[MINCLE]_) of cells from eight independent blood donors. **(B)** Reduced expression in DC is restricted to MINCLE, MCL, DECTIN2, and CLEC12A. Gene expression of human GM-CSF-elicited macrophages was compared to dendritic cells (induced *via* GM-CSF + IL-4) after 6 days of differentiation using qPCR. The data are depicted as mean ± SD of ΔΔCT values (using GM-CSF Mϕ as calibrators) from 6 independent blood donors. **(C)** IL-4 is sufficient to reduce MINCLE expression in human CD14^+^ cells. CD14^+^ peripheral blood mononuclear cells were stimulated as indicated. MINCLE expression was determined using qPCR. The data are depicted as mean ± SD (*n* = 2 for d0, *n* = 4 for d0.25, and *n* = 6 for all other time points). Asterisks indicate significant *p*-values for the comparison “IL-4 vs. untreated”, crosses for the comparison “GM-CSF + IL-4 vs. GM-CSF”. **(D)** Dose-dependence of IL-4 effect. CD14^+^ peripheral blood mononuclear cells were stimulated with increasing concentrations of IL-4 for 24 h. Mean ± SD of ΔCT values for MINCLE expression of cells from four independent donors. **(E)** Human CD14^+^ monocyte-derived M-CSF or GM-CSF elicited macrophages and dendritic cells were stimulated with IL-4 or left untreated for 48 h. Subsequently, MINCLE expression was determined using qPCR. The data are depicted as mean ± SD of cells from four independent blood donors. Statistical significance was tested using a paired *t*-test. *p < 0.05, **p < 0.01, ***p < 0.001, ^†††^*p* < 0.001.

In our recent characterization of CLR expression on primary human antigen-presenting cells, we observed higher expression of MINCLE mRNA in macrophages generated with M-CSF or GM-CSF compared to DC differentiated in the presence of GM-CSF plus IL-4 (28). This effect was confirmed in independent experiments using cells from unrelated healthy donors, showing a strong reduction in the ΔCT values from around −4.05 (M-CSF) and −5.0 (GM-CSF) to −10.4 (GM-CSF + IL-4) (Figure 1A). Quantitative RT-PCR analysis of mRNA expression for a range of CLR showed that the reduced expression in DC compared to GM-CSF-derived macrophages was selectively observed for MINCLE, MCL, DECTIN2, and CLEC12A whereas expression levels of the other tested CLR were not significantly different (Figure 1B). Since the presence of IL-4 in the cell culture media was the main difference in the differentiation protocols for GM-CSF macrophages and DC, we tested whether IL-4 alone would be sufficient to downregulate MINCLE expression in monocyte cultures (Figure 1C). MINCLE expression was highest in freshly isolated CD14^+^ monocytes and declined over time during culture in media alone. However, addition of IL-4 caused a stronger reduction, and this effect was detectable at all time points tested (6–144 h). The presence of GM-CSF kept MINCLE expression at a high level, but additional IL-4 induced strong downregulation, which was again detectable already after 6h, but increased over time (Figure 1C). The inhibitory effect of IL-4 on MINCLE expression by monocytes was dose dependent and already detected with 0.25 U/ml IL-4 but more pronounced at concentrations above 2.5 U/ml (Figure 1D). Having shown that IL-4 inhibits MINCLE expression in monocytes early during differentiation cultures, we next asked whether the same effect was seen when adding IL-4 to macrophages or DC after differentiation. While the expression of MINCLE in monocyte-derived DC cultured in media only was relatively variable and the reduction observed for the average values in DC treated with IL-4 was not significant, both types of macrophages responded with significant downregulation of MINCLE when treated with IL-4 for 48 h (Figure 1E).

### IL-4 also Downregulates Mincle Expression in Murine Antigen-Presenting Cells

**Figure 2:**
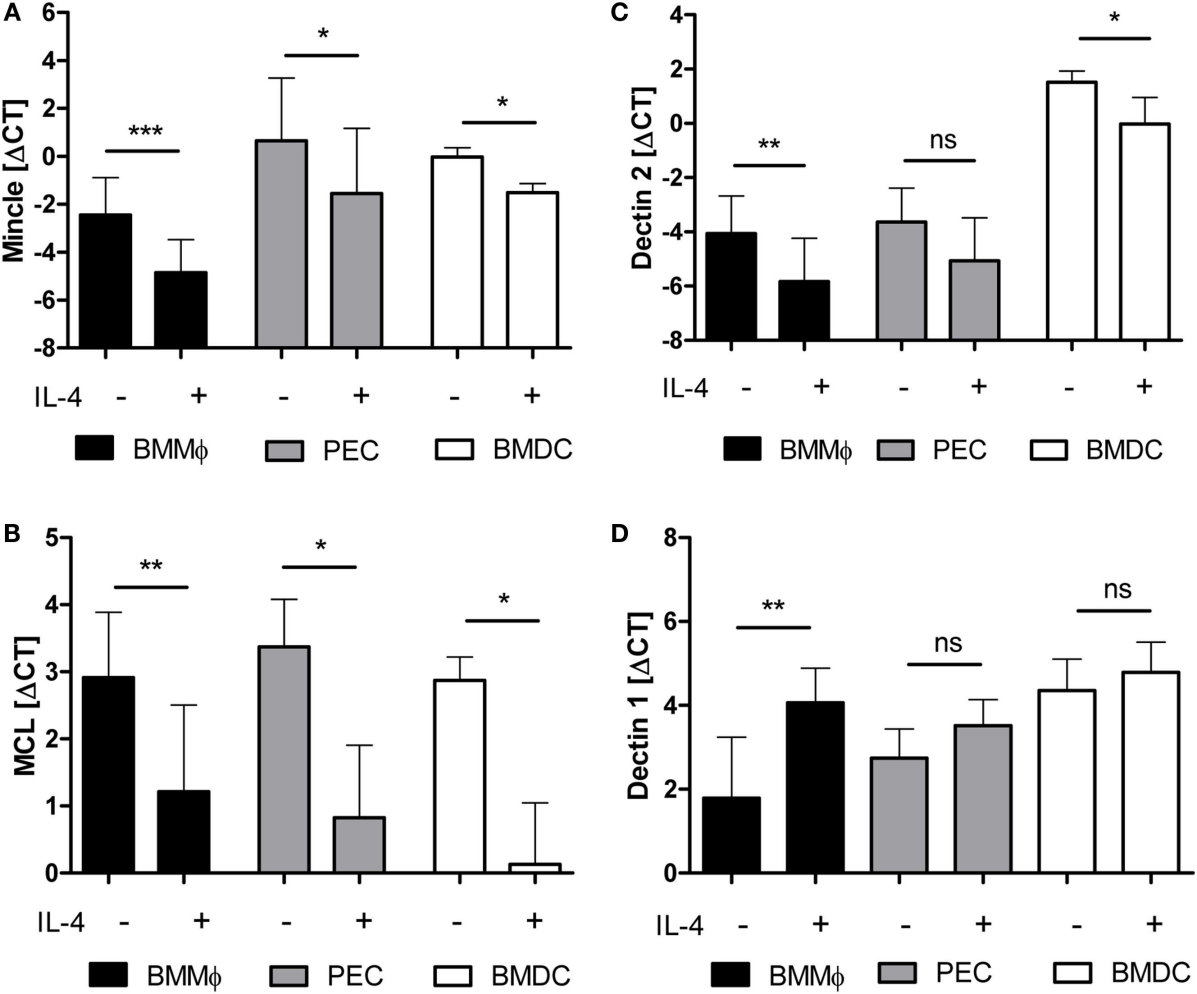
**mRNA expression of Mincle, Mcl, and Dectin-2 is downregulated by IL-4 on murine antigen-presenting cells**. Murine bone marrow-derived macrophages (BMM, black bars), thioglycolate-elicited peritoneal macrophages (PEC, gray bars), or bone marrow-derived dendritic cells (BMDC, white bars) treated or not with IL-4 for 48 h were analyzed by qRT-PCR for expression of mRNA encoding Mincle **(A)**, Mcl **(B)**, Dectin-2 **(C)**, or Dectin-1 **(D)**. Mean ± SD of ΔCT values from eight (BMM) or three (PEC and BMDC) independent experiments. Statistical significance was tested using a paired *t*-test. *p < 0.05, **p < 0.01, ***p < 0.001.

We next asked whether the regulatory action of IL-4 is conserved between species and tested the expression of mouse macrophages and DC treated for 48 h with IL-4 (Figure 2). IL-4 was used at the saturating concentration of 10 ng/ml (39). Mincle mRNA levels in M-CSF-driven BMM under basal conditions were lower than those in d4 thioglycollate-elicited peritoneal exsudate cells (PEC) or GM-CSF-driven BMDC, but IL-4 decreased Mincle expression in all three cell types significantly (Figure 2A). Mcl expression was comparably high in untreated BMM, PEC, and BMDC and strongly inhibited by IL-4 (Figure 2B). Dectin-2 mRNA was highest in BMDC and significantly downregulated by IL-4 in BMM and BMDC (Figure 2C). In contrast, IL-4 did not inhibit Dectin-1 expression in mouse APC, but in fact increased Dectin-1 mRNA in BMM, confirming a previous report (34) (Figure 2D). Together, the pattern of IL-4 effects on expression of the CLR family members examined was very similar between mouse and human, with significant downregulation of Mincle, Mcl, and Dectin-2, whereas Dectin-1 was not affected or even upregulated by IL-4.

### Differential Regulation of TDB- or LPS-Inducible Mincle Expression by IL-4

**Figure 3:**
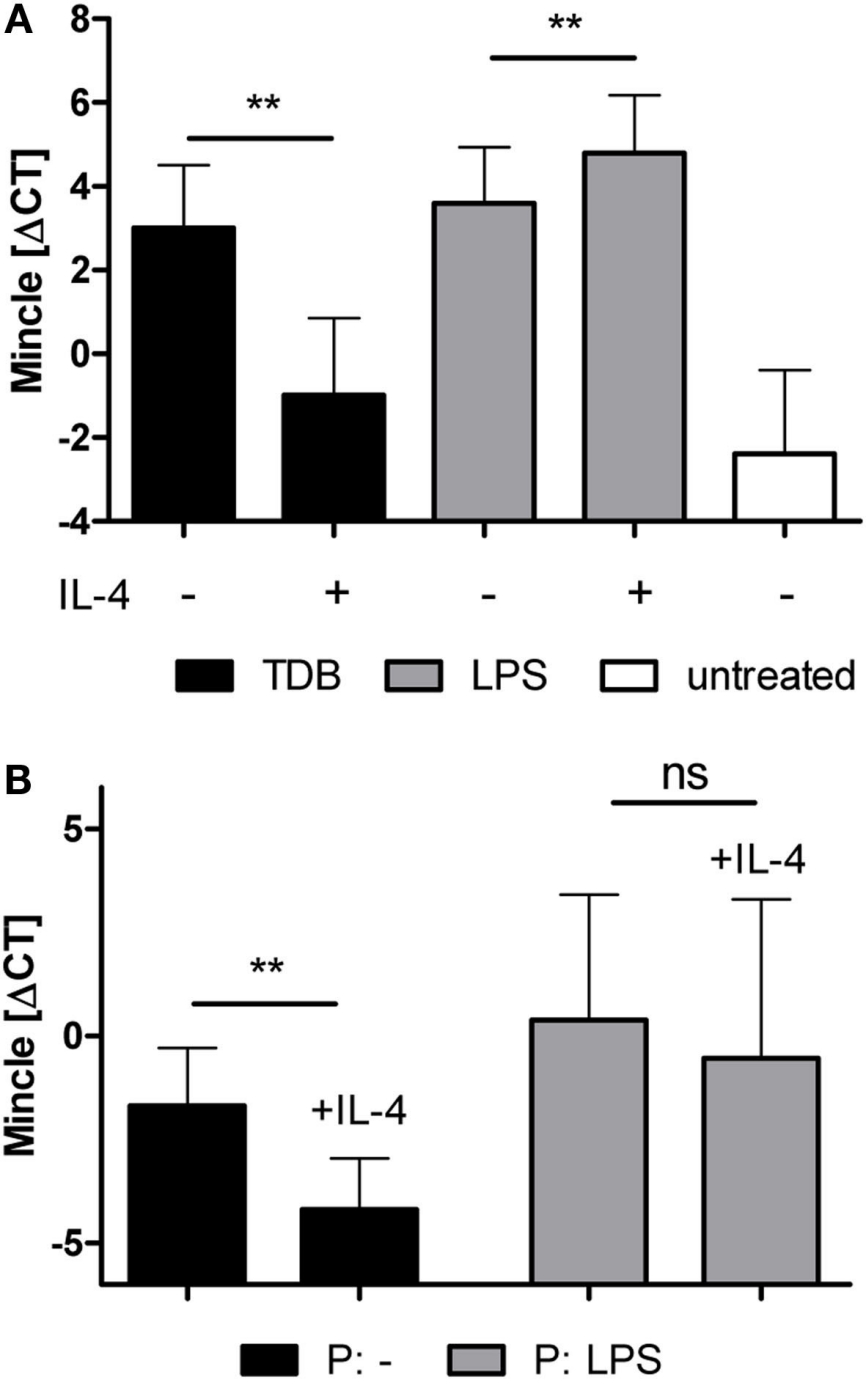
**LPS overcomes IL-4-induced downregulation of Mincle mRNA expression**. **(A)** Murine bone marrow-derived macrophages were treated with TDB or LPS, with or without IL-4, or were left untreated for 48 h. **(B)** LPS-priming abrogates the effect of IL-4 on Mincle expression. Bone marrow-derived murine macrophages were primed overnight with LPS *(P: LPS)* or left untreated *(P: -)*. The next day, LPS was washed off, the cells were re-plated and cultivated for 48 h with or without IL-4. Mincle expression was determined with qPCR. The data are depicted as mean ± SD of ΔCT values from five independent experiments. Statistical significance was tested using a paired *t*-test. **p < 0.01.

Expression of Mincle is strongly inducible by pattern recognition receptor signaling, such as TLR or CLR (4, 25, 40). Therefore, we examined the effect of IL-4 on Mincle mRNA expression in BMM treated with the TLR4 ligand LPS or the Mincle ligand TDB (Figure 3). Stimulation with LPS or TDB for 48 h significantly induced Mincle expression, as expected; the concomitant exposure to IL-4 inhibited Mincle upregulation by TDB, but not by LPS (Figure 3A). Priming of BMM with LPS or LPS + IL-4 overnight, followed by removal of LPS and washing before addition of IL-4, also abrogated the inhibitory effect of IL-4 on Mincle expression (Figure 3B). Thus, the inhibitory effect of IL-4 on Mincle expression could be overcome by stimulation of macrophages with the TLR4 ligand LPS.

### IL-4 Abrogates Cell Surface Expression of Mincle and Mcl Protein in Response to TDB but Not LPS

**Figure 4:**
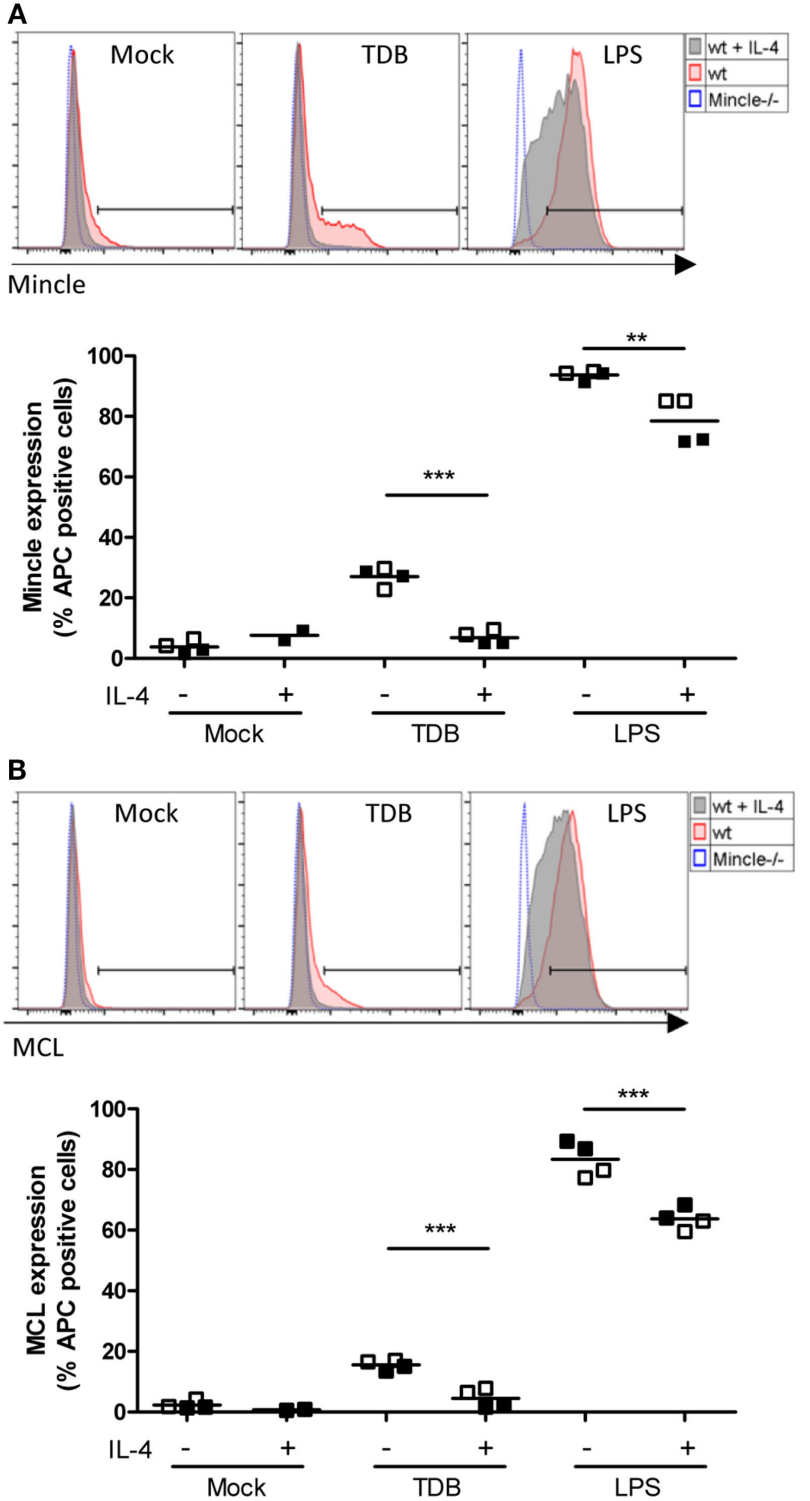
**Regulation of cell surface levels of Mincle and Mcl in murine macrophages by IL-4**. Bone marrow-derived macrophages were analyzed for Mincle **(A)** and Mcl **(B)** surface expression 16 h after stimulation as indicated by using anti-Mincle (4A9) or anti-Mcl (3A4) primary antibodies followed by staining with APC-conjugated secondary antibody. **(A)** Histograms show Mincle surface expression in C57BL/6 BMMs (wt) stimulated with TDB or LPS or left untreated (Mock) in presence (gray filled) or absence (red filled) of IL-4 as indicated. Mincle−/− BMMs (blue dotted) were stimulated with TDB or LPS or left untreated and used as negative control. Histograms are from one representative experiment. Scatter plot shows Mincle expression depicted as percentage of APC-positive cells from C57BL/6 BMMs after stimulation with TDB or LPS in presence or absence of IL-4 or left untreated (Mock). Data are depicted from two independent experiments performed in duplicates (filled symbols: exp. 1, open symbols: exp. 2). **(B)** Histograms and scatter plot show Mcl surface expression, respectively. Cells were treated as described in **(A)**. Statistical significance was tested using an unpaired *t*-test. **p < 0.01, ***p < 0.001.

We next employed flow cytometry to determine the cell surface levels of Mincle and Mcl in resting and stimulated macrophages (Figures 4A,B). Confirming previous reports (40, 41), only weak staining with the Mincle- and Mcl-specific monoclonal antibodies (4A9 and 3A4) was detected in resting macrophages. In fact, using Mincle-deficient macrophages as staining controls, only a minimal increase in the fluorescence signal for Mincle was observed. Overnight stimulation with TDB caused upregulation of Mincle and Mcl on a significant fraction of the macrophage population. However, this increase was almost completely prevented by co-treatment with IL-4. In contrast, the LPS-induced surface staining for Mincle and Mcl was found on the entire macrophage population and was only weakly reduced in the presence of IL-4. Together, the changes in cell surface protein levels and mRNA expression of Mincle and Mcl showed a close correlation.

### IL-4 Inhibits G-CSF and TNF Expression and Secretion by Macrophages in Response to TDB

**Figure 5:**
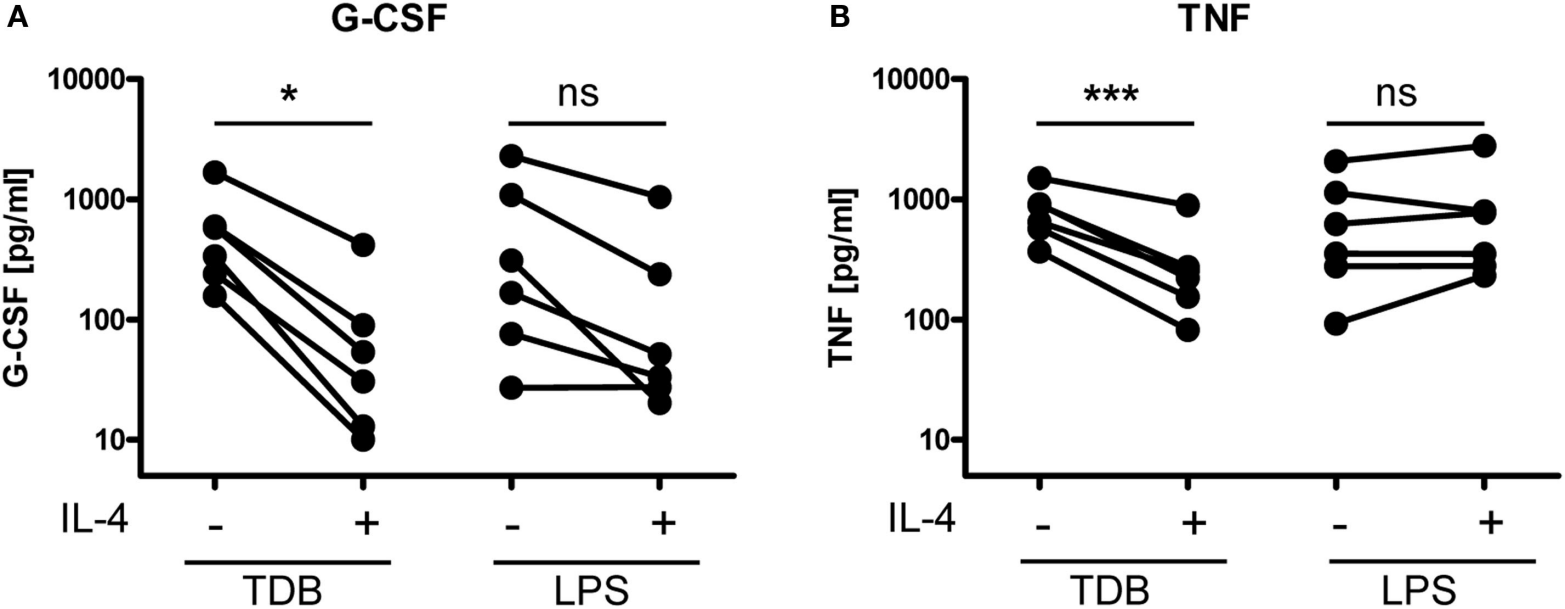
**IL-4 inhibits TDB-induced cytokine production in murine macrophages**. Bone marrow-derived macrophages were stimulated for 24 h with TDB or LPS in the presence or absence of IL-4. Cytokine levels in cell culture supernatant were quantified using ELISA [**(A)**, G-CSF, and **(B)**, TNF]. **(A,B)** Depicted are mean ELISA values of replicates from six independent experiments. Supernatants of BMM treated with media or IL-4 alone did not contain significant amounts of G-CSF or TNF (data not shown). Statistical significance of the effect of IL-4 on stimulation with TDB and LPS, respectively, was tested using a two-sided paired *t*-test. *p < 0.05, ***p < 0.001.

We reasoned that inhibition of Mincle expression in macrophages treated with IL-4 may functionally impair the response to the Mincle ligand TDB. To test this hypothesis, we determined the levels of the cytokines G-CSF and TNF, which are robustly secreted by macrophages stimulated with TDB or with LPS although the levels varied considerably between independent experiments (Figures 5A,B). Macrophages treated with IL-4 or media alone did not produce significant amounts of G-CSF or TNF (data not shown). Concomitant treatment with IL-4 strongly and consistently inhibited the production of both cytokines in response to TDB (Figures 5A,B). In contrast, LPS-induced G-CSF was inhibited less strongly by IL-4 (Figure 5A), and the LPS-induced release of TNF was not affected by IL-4 (Figure 5B).

### Negative Regulation of Mincle Expression and Target Gene Expression by IL-4 Requires Stat6

**Figure 6:**
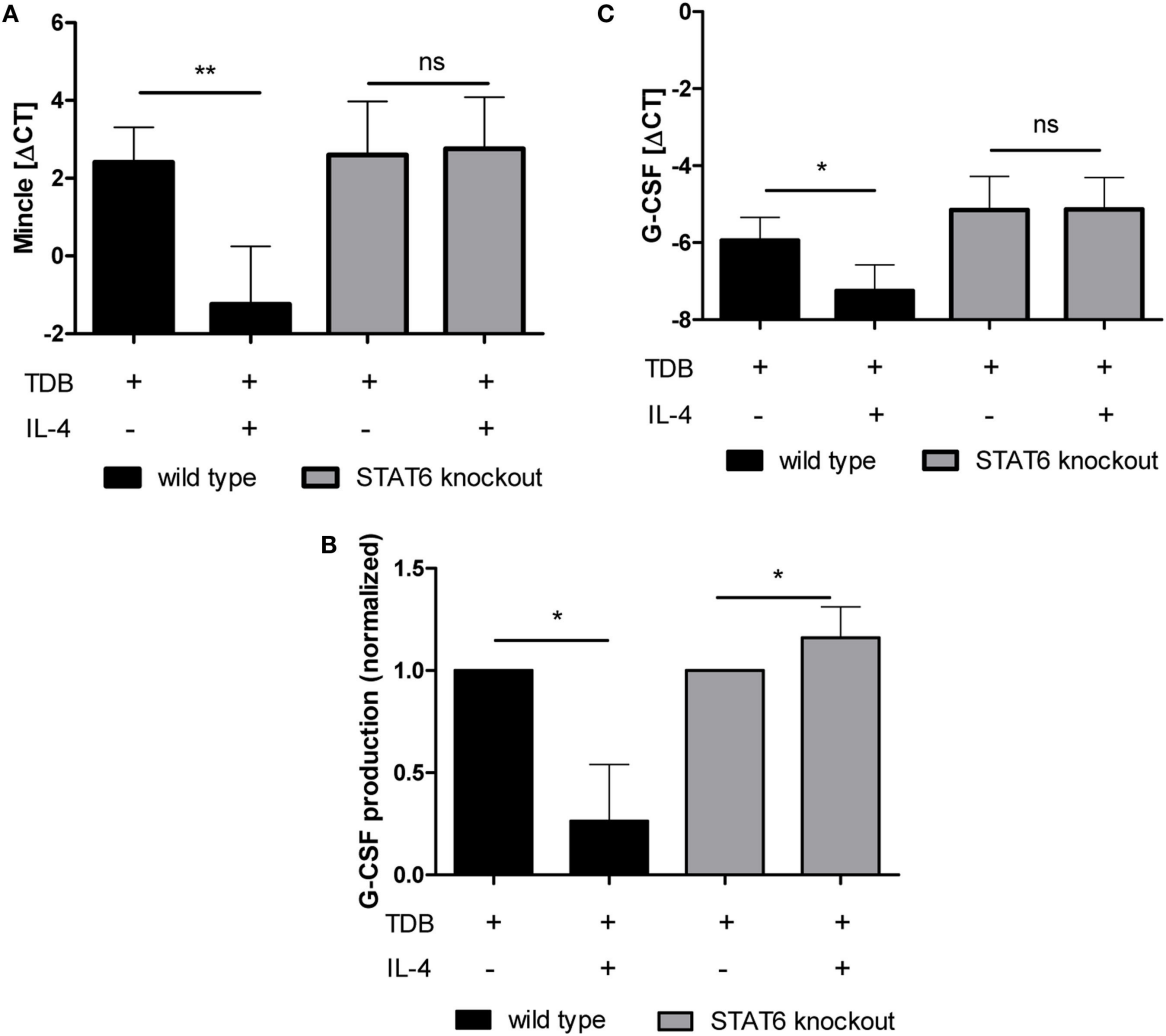
**IL-4-mediated inhibition of TDB-induced Mincle expression and G-CSF production is STAT6-dependent**. Bone marrow-derived macrophages from STAT6-deficient mice and Balb/c wild-type controls were stimulated for 48 h with TDB in the presence or absence of IL-4. Mincle and G-CSF expression was determined by qPCR **(A,C)**, and G-CSF concentration in the cell supernatant was obtained using ELISA **(B)**. ELISA values were normalized to TDB-induced G-CSF production. Mean ± SD of four independent experiments. Statistical significance was tested using a paired *t*-test. *p < 0.05, **p < 0.01.

IL-4 triggers Jak-Stat signaling, with Stat6 being the pivotal transcription factor for IL-4-induced gene expression (42). We investigated whether the inhibitory effect of IL-4 was also dependent on this transcription factor by using Stat6^−/−^ BMM in comparison to the respective Balb/c wild type control BMM (Figure 6). The Stat6 genotype had no effect on the basal (data not shown) or TDB-induced Mincle expression (Figure 6A). However, IL-4 inhibited the basal (data not shown) and TDB-induced Mincle expression in Balb/c BMM, but not in Stat6^−/−^ BMM (Figure 6A). Similarly, the TDB-inducible expression of G-CSF at the mRNA and protein level was strongly inhibited in Balb/c BMM, but not in Stat6^−/−^ BMM, where in fact a moderate increase in G-CSF protein secretion production was evident (Figures 6B,C).

## Discussion

Expression of pattern recognition receptors is a prerequisite for sensitive detection of microbial pathogens and is often restricted to specialized cell types of the innate immune system. While the CLR Mincle, Dectin-2, and Mcl are all expressed in myeloid cells, their expression levels are differentially affected by microbial and inflammatory stimuli. In this manuscript, we show that the prototypical Th2 cytokine IL-4 downregulates expression of these members of the Dectin-2 family, but not of other CLR, e.g., Dectin-1. Importantly, this inhibitory effect of IL-4 was conserved across species, as it was detected in human as well as murine APC. Reduced receptor expression may be functionally relevant, because production of cytokines in response to the Mincle ligand TDB was reduced in IL-4-treated macrophages. Our findings raise several questions concerning the mechanism and potential consequences of Dectin-2 family regulation by IL-4 during immune responses.

Signaling by the IL-4 receptor activates the transcription factor Stat6, which is required for most transcriptional responses (42). Therefore, the dependence of negative regulation of Mincle expression on Stat6 was not surprising. Further investigation will be required to determine in detail how Stat6 mediates directly or indirectly the decrease in Mincle mRNA expression. One possibility is that Stat6 interferes with the recruitment of activating transcription factors to the promoter/enhancer regions of Mincle, Mcl, and Dectin-2. The transcription factor C/EBPβ is essential for the inducible expression of Mincle, as shown already by Matsumoto et al. in 1999 (25), but also for upregulation of Dectin-2 and Mcl in response to TDB, whereas it is not required for Dectin-1 expression (26). Thus, the inhibitory effect of IL-4 and the requirement for C/EBPβ in inducible receptor expression correlate. It is conceivable that IL-4 may downregulate C/EBPβ at the transcriptional or post-transcriptional level in macrophages. Stat6 and C/EBPβ cooperate in the induction of several genes associated with Th2 responses (43) and alternative macrophage activation, such as Arginase-1 (44, 45). However, to our knowledge, no inhibition of C/EBPβ-dependent transcriptional responses by Stat6 has been described to date. To answer these questions, chromatin immunoprecipitation assays for direct binding of C/EBPβ and Stat6 to the promoter regions of Mincle, Mcl, and Dectin-2 will be required in future experiments.

Although IL-4 potently inhibited Mincle expression during differentiation of DC from human monocytes or in mouse macrophages, strikingly, the concurrent stimulation or priming of macrophages with the TLR4 ligand LPS overcame the IL-4 effect and prevented Mincle downregulation (Figure 3). It is possible that LPS interferes with IL-4 signaling through induction of negative regulators, such as Socs1 (46, 47). However, LPS did not block the induction of IL-4 induced Arginase-1 expression but rather synergistically induced it (data not shown), suggesting that the abrogation of the inhibitory IL-4 effect on Mincle expression likely involves other mechanisms. Regardless of the mechanism, it appears that TLR ligands make macrophages unresponsive to IL-4-triggered downregulation of Mincle expression. This effect would be consistent with the notion that the presence of TLR ligands signals the requirement for continued high level expression of CLR, regardless of other signals which otherwise would reduce their expression.

We have also shown here that macrophages treated with IL-4 not only express less Mincle but also fail to respond to TDB stimulation with upregulation of Mincle and Mcl (Figures 3 and 4), and of the cytokines G-CSF and TNF (Figure 5), indicating that reduced receptor expression may lead to weaker macrophage activation in response to mycobacterial cord factor. It remains to be tested in the future whether IL-4-induced downregulation of Mincle, Mcl, and Dectin-2 functionally impairs innate recognition of and the response to mycobacteria and fungi. From a technical point of view, our results also suggest that the use of growth factors and cytokines in protocols for generation of DC and macrophages, i.e. inclusion of IL-4 or not, may have strong effects on the response to CLR ligands. In the case of mycobacteria, the combined recognition of TDM by Mincle and Mcl, and of manLAM by Dectin-2, would be affected by IL-4, which may lead to compromised macrophage responsiveness to mycobacteria. It is interesting to speculate that in Th2-primed individuals reduced expression of Mincle, Mcl, and Dectin-2 on myeloid cells may occur, which could impair the detection of mycobacteria, and thereby could contribute to increased susceptibility to mycobacterial infection. Coinfection with helminths and *M. tuberculosis* is prevalent in many parts of the world. Epidemiological data show that exposure of household contacts to patients with active pulmonary TB resulted in higher risk to develop latent tuberculosis in the case of concurrent helminth infection, consistent with an impaired capacity of the innate immune cells, in this case alveolar macrophages, to kill mycobacteria upon inhalation (48). In addition, anti-helminthic treatment of people latently infected with *M. tuberculosis* led to marked improvement of T cell responses, which could also be at least partially due to upregulation of CLR expression with loss of helminth-induced IL-4 (49, 50). Indeed, such a mechanism appears to operate for levels of TLR2 and TLR9 (49). In the mouse model of coinfection, experimental evidence for increased susceptibility of helminth-infected animals challenged with *M. tuberculosis* has been obtained for increased mycobacterial burden for coinfection with *Schistosoma mansoni* or *Nippostrongylus brasiliensis* (32, 51). While helminth-induced IL-4 probably acts at multiple levels on the innate as well as the development of adaptive immune responses (52), the possible contribution of impaired expression of Mincle, Mcl, and Dectin-2 to modulation of the host response in tuberculosis in individuals co-infected with worms should therefore be explored in future studies.

## Author Contributions

TH, JS, and JO performed experiments and analyzed data. KJ performed experiments. DV provided critical reagents. JO and RL designed experiments and wrote the manuscript.

## Conflict of Interest Statement

The authors declare that the research was conducted in the absence of any commercial or financial relationships that could be construed as a potential conflict of interest.

## Abbreviations

CLR, C-type lectin receptor; FcRγ, Fc receptor gamma chain; IL-4, Interleukin-4; Mincle, macrophage-inducible-C-type-lectin; Mcl, macrophage C-type lectin; SYK, spleen tyrosine kinase; TDB, trehalose-6,6-dibehenate; TDM, trehalose-6,6-dimycolate.
